# Intracerebral inoculation of healthy non-transgenic rats with a single aliquot of oligomeric amyloid-β (1–42) profoundly and progressively alters brain function throughout life

**DOI:** 10.3389/fnagi.2024.1397901

**Published:** 2024-08-02

**Authors:** Marco Kramer, Thu-Huong Hoang, Honghong Yang, Olena Shchyglo, Juliane Böge, Ute Neubacher, Jens Colitti-Klausnitzer, Denise Manahan-Vaughan

**Affiliations:** Medical Faculty, Department of Neurophysiology, Ruhr University Bochum, Bochum, Germany

**Keywords:** rodent, amyloid-beta, amyloidosis, Alzheimer, synaptic plasticity, seeding

## Abstract

One of the puzzling aspects of sporadic Alzheimer’s disease (AD) is how it commences. Changes in one key brain peptide, amyloid-beta (Aβ), accompany disease progression, but whether this comprises a trigger or a consequence of AD is still a topic of debate. It is clear however that the cerebral presence of oligomeric Aβ (1–42) is a key factor in early AD-pathogenesis. Furthermore, treatment of rodent brains with oligomeric Aβ (1–42) either *in vitro* or *in vivo*, acutely impairs hippocampal synaptic plasticity, creating a link between Aβ-pathology and learning impairments. Here, we show that a once-off inoculation of the brains of healthy adult rats with oligomeric Aβ (1–42) exerts debilitating effects on the long-term viability of the hippocampus, one of the primary targets of AD. Changes are progressive: months after treatment, synaptic plasticity, neuronal firing and spatial learning are impaired and expression of plasticity-related proteins are changed, in the absence of amyloid plaques. Early changes relate to activation of microglia, whereas later changes are associated with a reconstruction of astroglial morphology. These data suggest that a disruption of Aβ homeostasis may suffice to trigger an irreversible cascade, underlying progressive loss of hippocampal function, that parallels the early stages of AD.

## 1 Introduction

Changes to the brain begin decades before the manifestation of Alzheimer’s disease (AD) (Jack et al., 2013). Early elevations in β-amyloid (Aβ) levels are an undisputed hallmark of (hereditary) familial Alzheimer’s disease (FAD) (Edwards, 2019). Aβ(1–42) in its oligomeric form is neurotoxic (Spires et al., 2005) and ultimately forms insoluble aggregates (plaques) that are one of the characteristics of AD (Edwards, 2019). Elevated levels of Aβ have been detected in cerebrospinal fluid decades before patients show AD symptoms (Fukumoto et al., 2010). Correspondingly, it has been proposed that the gradual accumulation of aggregated Aβ initiates a multistage cascade that eventually leads to AD (Hardy and Selkoe, 2002; Li and Selkoe, 2020). Sporadic AD has no known genetic basis, or clear etiology. Thus, one unanswered question is how sporadic AD gains a foothold in its early stages.

Known risk factors for sporadic AD comprise metabolic and homeostatic changes related to type 2 diabetes (Shieh et al., 2020), chronic inflammation (Paouri and Georgopoulos, 2019), obesity (Liu et al., 2020), chronic stress (Caruso et al., 2019), or sensory dysfunction (Lin et al., 2017; Dintica et al., 2019), suggesting that processes that place metabolic and neural homeostasis under an increased burden render the brain vulnerable to the propagation of oligomeric Aβ and consequently AD. In line with this, it has been suggested that impoverished Aβ clearance sets the stage for the pathogenesis of AD (Hardy and Selkoe, 2002; Li and Selkoe, 2020). In keeping with this possibility, others have reported that Aβ can serve as a seed for amyloidosis that propagates Aβ in transgenic mouse models of AD (Jucker and Walker, 2013). In this case, the introduction of very small quantities of Aβ into the brains of transgenic mice that overexpress amyloid precursor protein (APP), and/or express a gene encoding FAD, lead to propagation of Aβ (Kane et al., 2000; Katzmarski et al., 2020; Morales et al., 2021). Furthermore, it has been proposed that the development of AD in patients that were treated with human growth hormone may have derived from Aβ seeding through Aβ–contaminated hormone preparations (Jaunmuktane et al., 2015; Banerjee et al., 2024). In all of the abovementioned cases, a ‘pre-existing condition’ heralds the vulnerability to AD or supports the propagation of the seeding event.

Amyloidogenic Aβ peptides are generated from the membrane protein, APP: It contains a large N-terminal ectodomain and a small C-terminal cytoplasmic domain (Kang et al., 1987). Under normal circumstances, APP is cleaved by α-secretase into a soluble N-terminal fragment (sAPPα) and a membrane-bound C-terminal fragment (CTFα). Further cleavage by γ-secretase generates N-terminal fragments (p3) comprising Aβ (17–40) and Aβ (17–42), from CTFα (Esch et al., 1990; Lichtenthaler et al., 1997). Sequential proteolytic cleavage by β-secretase on the N-terminus and by γ-secretase on the C-terminus generates a soluble N-terminal fragment (sAPPβ), a membrane-bound C-terminal fragment (CTFβ) and amyloidogenic N-terminal fragments (p4) that correspond to Aβ (1–40) and Aβ (1–42) (Hardy and Selkoe, 2002). It is presumed that, under normal circumstances, neural housekeeping prevents accumulation of Aβ(1–40/42): for example, microglia and astrocytes are activated by the presence of these peptides and mediate their clearance (Meda et al., 1995; Deane et al., 2009; Ries and Sastre, 2016; Haque et al., 2018). It is likely however, that the pathophysiological cascade of AD begins when these processes are overwhelmed or are fundamentally altered by amyloidogenic forms of Aβ (Acosta et al., 2017).

How much Aβ is too much? It has been demonstrated that endogenous Aβ promotes and supports hippocampal synaptic plasticity, a key mechanism in memory formation (Puzzo et al., 2008, 2011) and effects are concentration-dependent and largely enabled by Aβ monomers (Gulisano et al., 2018). But the topical administration of Aβ to hippocampal slices (Walsh et al., 2002), or the acute application of oligomeric Aβ directly into the brains of rodents, inhibits LTP, alters hippocampal network dynamics and impairs cognition (Cleary et al., 2005; Walsh and Selkoe, 2007; Kalweit et al., 2015), suggesting that the presence of oligomeric Aβ(1–42) may be a first step toward pathological changes of the brain. Transgenic mice that overexpress APP, or possess other gene modifications that predispose toward AD, show progressive changes in brain health that reflect amyloidosis and AD symptoms (Kosel et al., 2022). To what extent Aβ seeding can lead to debilitating changes of the brain in individuals or animals that are ostensibly healthy and transgenically unmodified is, as yet, unknown. Clarification of this aspect would bring us a step forward in understanding how sporadic forms of AD can develop.

In this study we asked the question as to what extent the healthy brain can cope with a transient increase in oligomeric Aβ and, in particular, whether this has any lasting consequences for hippocampal function. We report here that a single intracerebral administration of oligomeric Aβ(1–42) to healthy adult rats triggers a progressive debilitation of brain function that becomes increasingly manifest over a period of months after treatment.

## 2 Materials and methods

### 2.1 Animals and treatment

The study was carried out in accordance with the European Communities Council Directive of September 22nd 2010 (2010 / 63 / EEC) for care of laboratory animals and in accordance with requirements of the animal ethics committee of the local government authority (Landesamt fur Arbeitsschutz, Naturschutz, Umweltschutz und Verbraucherschutz (LANUV), North-Rhine Westphalia). Male Wistar rats were used that were 7–8 weeks old at the time of initial Aβ–treatment. Animals underwent regular physical health checks to verify that they had a continuous clean bill of health throughout the entire study. Animals were group-housed in a temperature- and humidity-monitored vivarium with a constant 12-h light–dark cycle (lights on from 7 a.m. to 7 p.m.) with *ad libitum* food and water access. For the purpose of in vitro electrophysiology, or immunohistochemistry studies, rats were stereotaxically injected under sodium pentobarbital anesthesia (52 mg/kg, intraperitoneally, i.p.), with Aβ(1–42) oligomers, or scrambled (control) peptide into the lateral cerebral ventricle in a dose of 10 μM, applied in 5 μL, over a 5 min period via a Hamilton syringe (Kalweit et al., 2015). The injection coordinates comprised: 0.5 mm posterior to bregma, 1.6 mm lateral to midline, 5.6 mm depth from skull surface. Further experiments in vitro and immunohistochemistry assessments were conducted 1 week after treatment to assay for early hippocampal changes. Further experiments were conducted 1 month or 4–6 m onths after treatment (Supplementary Figure S1).

The oligomeric Aβ(1–42) peptide was generated as described previously (Bozso et al., 2010; Kalweit et al., 2015). Control peptide comprised a scrambled version of the Aβ(1–42) peptide [referred to here as ScrAβ(1–42)] that contains a permutated amino acid sequence (Bozso et al., 2010; Kalweit et al., 2015). Previous studies confirmed that this scrambled peptide has no significant effect on neuronal function (Südkamp et al., 2021). Before intracerebral treatments, the peptides were incubated in phosphate-buffered saline (PBS) at a pH of 7.4 for 3 h at a concentration of 50 μM and at room temperature to ensure adequate oligomerization of the Aβ(1–42) peptide (Bozso et al., 2010). The mixture was then diluted to the final concentration of 10 μM. This dose was equivalent to 50 pmol, or 225 ng of the respective peptide.

### 2.2 *In vivo* electrophysiological recordings and cannula implantation

Rats underwent implantation of electrodes into the medial perforant path and the dorsal dentate gyrus, under sodium pentobarbital anesthesia (Nembutal, 52 mg/kg, intraperitoneally (i.p.), Boehringer Ingelheim, Ingelheim, Germany), as described previously (Kalweit et al., 2015). During this procedure, a monopolar recording electrode was implanted in the suprapyramidal granule cell layer of dentate gyrus (3.1 mm posterior to bregma, 1.9 mm lateral to the midline) and a bipolar stimulation electrode was implanted in the medial perforant pathway (6.9 mm posterior to bregma, 4.1 mm lateral to the midline). To enable subsequent peptide treatment, a cannula was implanted into the lateral cerebral ventricle with the following coordinates: 0.5 mm posterior to bregma, 1.6 mm lateral to the midline. For *in vitro* electrophysiological and immunohistochemistry studies, only a cannula was implanted. Pre- and postsurgical analgesia was conducted using meloxicam (Metacam, 0.2 mg/kg, i.p., Boehringer Ingelheim Vetmedica GmbH, Ingelheim, Germany). Following verification of the integrity of the evoked potentials 1 week after surgery, the first set of experiments were commenced.

*In vivo* electrophysiological recordings of local field potentials were obtained from freely behaving rats. For this, an evoked response was triggered by stimulating at low frequency (0.025 Hz) with single biphasic square wave pulses of 0.2 ms duration per half wave, generated by a constant current isolation unit. For each time-point measured during the experiments, five records of evoked responses were averaged. The first 6 time-points recorded at 5 min intervals were used as baseline and all time-points are shown in relation to the average of these 6 points. The evoked potential manifested itself as a positive going field excitatory postsynaptic potential (fEPSP), upon which a negative-going population spike (PS) was imposed. The fEPSP was measured as the slope measured on the first five steepest point of the positive-going potential. The PS was measured as the amplitude obtained on the first negative deflection of the PS. The direction of change of fEPSP and PS was consistently the same (not shown). By means of a stimulus–response determination (evaluation of nine different stimulation intensities from 100 to 900 μA in 100 μA steps) the maximal PS amplitude was found, and during experiments all potentials employed as baseline criteria were evoked at a stimulus intensity that produced 40% of this maximum. Long-term potentiation (LTP) was evoked by means of high-frequency stimulation (HFS, comprised of a stimulus burst of 15 pulses each of 0.2 ms duration applied at 200 Hz and repeated 10 times with a 10 s interval between bursts). For each time-point 5 consecutive evoked responses at 40 s intervals were averaged, and the results were expressed as the mean percentage ± standard error of the mean (S.E.M.) of the average of the first 6 recordings. Recordings were made every 5 min. Until 30 min after HFS and then every 15 min until 4 h had elapsed. The following day an additional 1 h of recordings was obtained. Peptide treatment occured after the initial verification of LTP.

Postmortem analysis of electrode localization was conducted to verify the final location of the electrodes. The brains were carefully removed from the cranium, the tissue was fixed in 4% paraformaldehyde (PFA) in phosphate buffered saline (PBS, pH 7.4) for 5–7 days following by cryoprotection in 30% sucrose. The brain tissue was then cut frontally on a cryostat (Leica CM 3050S) into 30 μm slices. After mounting on gelatin-coated slides, the sections were stained in 0.1% cresyl violet (Sigma-Aldrich Chemie GmbH, Munich, Germany). This staining was conducted in order to visualize the hippocampal structures and identify the electrode tracts. Data from animals with misplaced electrodes were excluded from the study.

### 2.3 *In vitro* electrophysiological recordings

Two groups of rats were treated intracerebrally with oligomeric Aβ(1–42), or control peptide (ScrAβ(1–42)), for *in vitro* electrophysiological studies. The first group was investigated 1 week after injection, and the second after 4–6 months. The range of 4–6 months was chosen for patch clamp experiments because of the increasing difficulty of obtaining effective patch seals on cells from the aging hippocampus (Ting et al., 2014). Animals were deeply anesthetized by inhalation of the anesthetic isoflurane, and then decapitated. The brains were rapidly removed and dissected in ice-cold dissection medium composed of (in mM): NaCl (87), KCl (2.4), MgSO_4_ (1.3), CaCl_2_ (0.5), NaHCO_3_ (26), NaH_2_PO_4_ (1.25), D-Glucose (2). Transverse hippocampal slices (350 μm thick) were prepared using a vibrating blade microtome (VTS1000, Leica, Germany). After cutting, the slices were incubated in a holding chamber in the dissection medium for 30 min at 35°C. After incubation, the slices were transferred to recording chambers where they were used for patch-clamp recordings. During experiments, the slices were continuously perfused with oxygenated (95% O_2_/ 5%CO_2_) artificial cerebrospinal fluid (aCSF) of the following composition (in mM): NaCl (125), KCl (3), MgSO_4_(1.3), CaCl_2_(2.5), NaHCO_3_(26), NaH_2_PO_4_(1.25), D-Glucose (13). The flow rate of aCSF was 1.5–2 mL/min. The temperature in the recording chamber was maintained at 30°C. With the help of an upright BX51WI microscope (Olympus, Japan), whole-cell patch clamp recordings were then performed from visually identified cell bodies of dentate gyrus granule cells using infrared illumination.

Borosilicate glass recording pipettes, used for recordings, were filled with an intracellular solution comprising (in mM): Potassium gluconate (97.5), KCl (32.5), EGTA (5), HEPES(10), MgCl_2_(1), Na_2_ATP(4) (pH 7.3; 290 mOsm). Recordings were performed in current-clamp mode using an amplifier (EPC10 USB, HEKA Electronic, Germany). The data were subjected to low-pass filtering at 2.9 kHz and digitized at 10 kHz.

Intrinsic membrane properties were analyzed using PATCHMASTER acquisition software and AP feature software (MATLAB code developed in Department of Psychology, University of Connecticut, courtesy of Prof. M. Volgushev). Resting membrane potential was determined as the mean value recorded during a continuous period of 10 s. Input resistance was calculated from the slope of the linear fit of the relationship between the change in membrane potential (∆V) and the intensity of the applied current (between −60 pA and + 60 pA) duration 600 ms. To analyze action potential properties, square currents (duration 600 ms) were applied in the range of 5pA to 300pA, using 5pA steps. The amount of current necessary to evoke an action potential from resting membrane potential was determined as a threshold current in pA. The first action potential was subjected to analysis of its active properties, such as the maximum and minimum spike voltage, spike amplitude, action potential width (value measured at the point where the AP had reached 20% of its maximum) and afterhyperpolarisation (AHP) amplitude. The action potential amplitude was measured as voltage difference from the threshold value to spike peak. Firing frequency properties were examined by applying square current pulses (duration 1 s) in the range of 0 pA to 400 pA in 50 pA steps. The firing frequency was analyzed as the number of spikes elicited during applied current (duration 1 s).

### 2.4 Behavioral tasks

Behavioral assessments were conducted in different groups of animals either 1 or 6 months following intracerebral inoculation with oligomeric Aβ(1–42), or control peptide. Behavioral tasks were conducted in an open field arena (80 × 80 × 80 cm). A camera was mounted above the open field to record the experiments for off-line analysis. Exploration of an object was defined as when the rat directly contacted the object with the snout, or with the snout pointed toward the object and was within 2 cm distance of the object.

**Figure 1:**
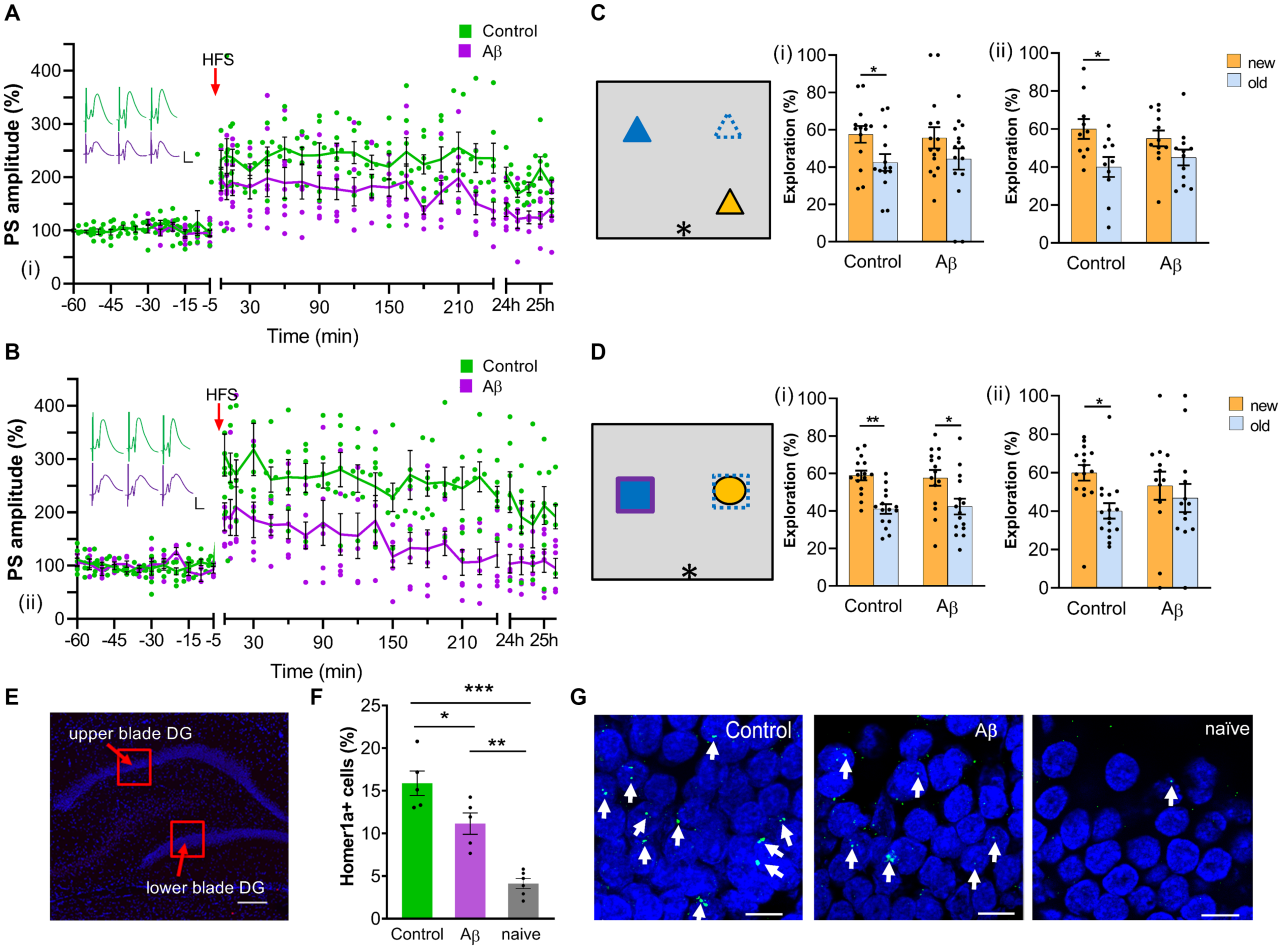
Progressive impairment of LTP 1 month and 6 months after Aβ-treatment. **(A)** One month after an intracerebral treatment with oligomeric Aβ (1–42) (*n* = 7), LTP was significantly reduced compared with age-matched (scrambled peptide-treated) controls (*n* = 7) (i). ANOVA: *F* (1,12) = 7.33, *p* < 0.05. Insets show analog traces of field potentials from one control (green) and one Aβ-treated rat (purple) recorded 5 min before (left), 5 min after (middle) and 24 h after (right) high-frequency stimulation (HFS). Horizontal scale bar: 5 ms, vertical scale bar: 5 mV. **(B)** Six months after Aβ-treatment, LTP was still impaired (Aβ: *n* = 6; controls: *n* = 7) (ii). ANOVA: *F* (1, 11) = 10.53, *p* < 0.01. Insets show analog traces of field potentials from one control (green) and one Aβ-treated rat (purple) recorded 5 min before (left), 5 min after (middle) and 24 h after (right) high-frequency stimulation (HFS). Horizontal scale bar: 5 ms, vertical scale bar: 5 mV. **(C)** Left: Schematic summary of the spatial recognition memory task. (i): one month after Aβ-treatment, rats (*n* = 13) explored objects at new and old locations equally, indicating impaired memory; whereas control (scrambled peptide-treated) rats (*n* = 14) explored relocated objects significantly longer. (ii): Animals showed impaired spatial recognition memory 6 months following Aβ-treatment (Aβ: *n* = 12; control: *n* = 10), *t*-test, *, *p* < 0.05. **(D)** Left: Schematic summary of the object recognition memory task. (i): One month after treatment, both control (*n* = 15) and Aβ-treated (*n* = 15) rats explored the new object significantly more than the old object. (ii): 6 months after the Aβ-treatment, object recognition memory was impaired (Aβ: *n* = 11; control: *n* = 14), *t*-test, *, *p* < 0.05. **(E)** Photomicrograph of areas scrutinized for somatic Homer1a expression in the suprapyramidal (upper) and infrapyramidal (lower) blades of the dentate gyrus 1 month after Aβ-treatment. The image shows DAPI –stained neurons of the dentate gyrus. Regions of interest in the upper and lower blades (red squares) were examined for somatic Homer1a expression after item-place learning. Scale bar: 200 μm. Images were acquired using a slide scanner at final magnification of 10x. **(F)** Exploration of item-place significantly enhances somatic Homer1a mRNA expression in the dentate gyrus of control (scrambled peptide-treated) animals compared to exploration-naïve animals (*n* = 6) [one-way ANOVA, *F*(2,13) = 30.533, *p* = 0.0000]. One month after Aβ-treatment (*n* = 5), Aβ-animals showed significantly reduced somatic Homer1a mRNA expression in the dentate gyrus after a novel spatial exploration task compared to controls (*n* = 5) (Tukey post-hoc test: control vs. naïve: ***p < 0.001, Aβ vs. naïve: **p < 0.01, control vs. Aβ: *p < 0.05). Effects were significant from exploration-naive animals indicating that experience-dependent Homer1a expression occurred in Aβ-treated animals. **(G)** Example of nuclear Homer1a expression in dentate gyrus granule cells (green dot, indicated by white filled arrows) of one control (left), one Aβ-treated rat (middle) and one naïve rat (right). The nuclei were stained in blue using DAPI. Scale bars: 10 μm. Pictures were taken using a 63x objective (Zeiss ApoTome). Data points from individual animals are superimposed upon the graphs and bar charts.

The rats were habituated to the testing procedure and allowed 10 min of exploration time in the empty open field for the 3 consecutive days preceding the experiment. After habituation, the rats were tested in a spatial recognition task (Figure 1C). In the sample trial, the rat was placed at the center of the open field with two identical objects (blue triangles in Figure 1C). After 5 min the rat was returned to its home box for a pause of 3 h before the test trial. In the test trial, the rat was placed at the center of the open field with the same two objects: one remained at the old location (blue filled triangle in Figure 1C), the other was displaced to a new location (orange filled triangle in Figure 1C). The new and old locations were randomized to avoid any possible preference of the location in the open field. The scorer was blind to the treatment the rats received.

The day after the spatial recognition task, the rats were tested in an object recognition task (Figure 1D). The experimental setup was as described above but used another two sets of novel objects. In the sample trial, the rat was placed at the center of the open field with two identical objects (blue rectangle in Figure 1D). After 5 min the rat was returned to its box for a pause of 3 h before the test trial. In the test trial, the rat was placed at the center of the open field with one old object (blue rectangle in Figure 1D) presented in the sample trial and one new object (orange oval in Figure 1D) and was allowed to explore for 5 min. The objects used as new or old objects were randomized and the location of new object was randomized. The scorer was blind to both the treatment of the rats and whether the object was new or old.

The exploration time of different objects was referred to as a percentage of the sum exploration time. Statistical analysis was conducted between the exploration time of different objects within one group of animals within one experiment.

### 2.5 Fluorescence *in situ* hybridization triggered by learning

In a different group of animals, fluorescence *in situ* hybridization (FISH) was conducted 1 month following intracerebral inoculation with oligomeric Aβ(1–42), or control peptide. Rats were handled by the experimenter and habituated to the recording chamber that measured (40 × 40 × 40 cm) for 3 consecutive days. On the experiment day, in the same chamber, the animals were exposed to two novel objects for 5 min followed by a pause of 3 h duration. In a subsequent exploration period, object A was returned to the chamber in exactly the same position as before, and object B was placed in a new location in the chamber (as shown in Figure 1D and described above). The animals explored for 5 min. During the exploration period, the behavior of the animals was recorded. The brains were rapidly removed 40 min after commencement of the second exploration and then shock-frozen in isopentane at −80°C on dry ice and stored at −80°C until being sectioned 20 μm thick on a Cryostat (Leica CM 3050S). The coronal sections containing hippocampus (3.6–4.0 mm posterior to Bregma) were mounted directly on ‘superfrost plus’ slides (Gerhard Menzel GmbH, Braunschweig, Germany) and stored at −80°C until further processing. We additionally included naïve animals that underwent the same handling and habituation procedure, as was conducted with the experimental groups. These animals did not undergo surgery. On the day of the experiment, they resided in the recording chamber for the same duration as the ‚item-place exploration’ animals until brain extraction, without engaging in an exploration task.

Fluorescein-labeled RNA probes were generated using the Ambion MaxiScript Kit (Invitrogen, Carlsberg, United States). Homer1a cDNA plasmid was prepared by Entelechon (Bad Abbach, Germany) and GenScript (GenScript Biotech, New Jersey, United States) using a 1.2 kb Homer 1a transcript according to the sequence of Brakeman et al. (1997). The cDNA probes were prepared from the linearized cDNA using Ambion MaxiScript Kit and premixed RNA labeling nucleotide mix containing the fluorescein-labeled UTP (Invitrogen, Carlsberg, United States). The RNA probes were purified on Mini Quick Spin RNA columns (Roche Diagnostics, Mannheim, Germany) and their yield and integrity were verified using gel electrophoresis.

At the time-point of further processing, one glass slide per animal (including three brain slices each) was chosen and left at room temperature until the slices were defrosted. The glass slide showing slices from the dorsal hippocampus at ~3.8 mm from Bregma was chosen for each individual animal. We then applied the following protocol for the Homer1a mRNA *in situ* hybridization (Hoang et al., 2018):

After 10 min of fixation in fresh, filtered and ice cold 4% PFA in PBS, the slides were washed for 2 min in 2x saline-sodium citrate buffer (SSC). The slides were then left for 10 min in acetic anhydride solution followed by 5 rinses in 2x SSC. After a final wash in 2x SSC for 5 min, the slides were prehybridized in formamide /4 x SSC solution (1:1) for 10 min at 37°C. The fluorescein-labeled RNA probe was diluted in 1x hybridization buffer (Sigma-Aldrich Chemie GmbH, Munich, Germany) (100 ng/100 μL), heated at 90°C for 5 min and the slides were hybridized for approximately 17 h in a humid chamber at 56°C. To confirm the specificity of the hybridized signal, we additionally conducted a negative control FISH test whereby the fluorescein labeled Homer1a RNA was not added to the brain sections (data not shown). Following the hybridization, the slides were first washed 3 × 5 min using 2 x SSC and the remaining single stranded mRNA was removed using 10 μg/mL RNase A in 2 x SSC for 15 min at 37°C. Afterwards, various washing steps were conducted: 10 min in 2x SSC at 37°C, 10 min in 0.5 x SSC at 56°C, 30 min in 0.5 x SSC at 56°C,10 min in 0.5 x SSC at room temperature (RT), 5 min in 1x SSC at RT twice, and finally 5 min in tris-buffered saline (TBS) thrice at room temperature. After these washing steps, we pretreated the slices with 3% H_2_O_2_ in TBS for 15 min followed by 3 exposures for 5 min to 1x TBS. In order to prevent unspecific binding of proteins we used 10% n-goat serum in TBS-Tween 20 (Polysorbate, 0.2% Tween, TBS-T) containing 20% Streptavidin (Vector Labs, Burlingame, USA) for 60 min followed by 90 min incubation with anti-fluorescein [1:2000] (Dianova, Hamburg, Germany) in 10% n-goat serum in TBS-T containing 20% Biotin (Vector Labs, Burlingame, USA). Following three washes, each of 5 duration, in TBS the signal was amplified using biotinylated Tyramine (bT. Adams, 1992). The slides were left in TBS containing bT (1%) and H_2_O_2_ for 20 min and afterwards washed three times for 5 min in TBS. Finally, we visualized the signal using StrepAvidin Cy2 (1:250) (Dianova, Hamburg, Germany) in 10% n-goat serum in TBS-Tween 20 at room temperature for 60 min. The slides were washed again 3 × 5 min in TBS, and quickly washed with distilled water. Afterwards, slides were quickly dipped in 70% ethanol, and stained using 1% Sudan black B (Merck KGaA, Darmstadt, Germany) in 70% ethanol (Oliveira et al., 2010). Slides were air-dried overnight and mounted in DAPI (4′,6-diamidino-2-phenylindole) containing Mounting medium (immunoSelect® Dianova, Hamburg, Germany).

For data analysis, we focused on changes in Homer1a expression in the soma of dentate gyrus granule cells, given that this is the region from which electrophysiological recordings were obtained in both the *in vitro* patch clamp and the *in vivo* synaptic plasticity experiments. We looked for Homer1a mRNA expression within the nuclei of the granule cells by obtaining z-stacks at a 63x magnification using a Zeiss ApoTome (Figure 1E). Three consecutive slices of each animal were used for the analysis, whereby we analyzed one hemisphere of each slice and calculated the mean of these three slices. Using Fiji software (Schindelin et al., 2012) the complete DAPI stained nuclei that were not cut on the edges either in the x, y or z direction, were marked. Afterwards, they were checked for Homer1a mRNA expression that peak in the nuclei of the granule neurons and the percentages of the Homer1a mRNA positive cells were calculated per total counted neurons for each subregion of each rat. The designation “positive nuclei” was given to cells that contained intense intranuclear-foci of Homer1a mRNA fluorescent signals (Figure 1G, green dots). Nuclei that did not contain any intranuclear-foci representing a fluorescent signal of Homer1a mRNA were counted as negative. The total number of dentate gyrus granule cells analyzed for each brain slide of each animal ranged from 80 to 100 in the upper (suprapyramidal) blade and, from 70 to 90 in the lower (infrapyramidal) blade. Then the averaged values from both blades of the dentate gyrus were calculated for each animal. Final results are presented as average mean of percentages ± SEM for each group. The analysis was conducted without the experimenter knowing the identity of the different animal groups.

### 2.6 Immunohistochemistry

For the purpose of immunohistochemical analysis, another two groups of rats were treated intracerebrally with oligomeric Aβ(1–42) (*n* = 16), or control peptide (*n* = 16) and sacrificed either 1 week or 6 months following this procedure. Glia analysis was performed in a smaller group of animals (*N* = 16) as a pilot experiment. After transcardial perfusion with 4% paraformaldehyde (PFA), brains were carefully removed and further fixated in 4% PFA overnight, followed by cryoprotection in sucrose solution (30%) at 4°C. Immunostaining was performed on free-floating horizontal 30 μm sections. We used **Anti-Aβ1-16** primary antibodies (1:400, mouse monoclonal AB, SIG-39320, Covance Inc., United States) to detect Aβ plaque deposition. As a positive control we tested the brains of 9 month old transgenic mice that express a mutant amyloid precursor protein (tgAPP) that is known to result in significant plaque expression in the brains of the animals at this age (Sasaguri et al., 2017).

Anti-glial fibrillary acidic protein **(Anti-GFAP)** primary antibodies (AB, 1:2000, polyclonal rabbit AB, Z0334, DakoCytomation Denmark A/S, Denmark) were used to assess differences in astroglia cell population (Yang and Wang, 2015). Anti-ionized calcium-binding adapter molecule 1 **(Anti-Iba1)** primary antibodies (1:500, polyclonal rabbit AB, #019–19,741, Wako Pure Chemical Industries, Japan) were used to investigate possible differences in microglia cell populations (Ito et al., 1998) and **Anti-GluN1** primary antibodies (1:200, monoclonal mouse AB, 556308, BD Biosciences Pharmingen, United States) were used to determine N-methyl-D-aspartate receptor (NMDAR) expression. Sections from control- and Aβ-treated animals underwent masked randomization and were processed together in order to avoid variations in the processing of data sets.

Sections were treated with 0.3% H_2_O_2_ in PBS for 20 min in order to deactivate endogenous peroxidases. To reduce unspecific background staining, endogenous biotin and electrostatic loading proteins were blocked by 10% normal serum, 20% Avidin (Avidin/Biotin Blocking Kit SP-2001, Vector Labs, Burlingame, United States) in PBS containing 0.2% Triton X-100 (Tx, Sigma-Aldrich Chemie GmbH, Munich, Germany). Sections were then incubated overnight (12 to 20 h) in a solution containing 1% normal serum, 20% Biotin (Avidin/Biotin Blocking Kit SP-2001, Vector Labs, Burlingame, USA) and the respective primary antibody (AB) in PBS-Tx. Sections were then incubated in PBS-Tx solution containing 1% normal serum and the corresponding secondary AB (biotinylated goat-anti-rabbit BA-1000 or biotinylated horse-anti-mouse BA-2001, Vector Labs, Burlingame, United States) at a dilution of 1:500 for 90 min. To detect the antibody binding an ABC *elite* kit (Vectastain® Elite ABC Kit PK-6100, Vector Labs, Burlingame, USA) was applied at a dilution of 1:1000 for 90 min. The peroxidase binding was visualized by incubation in 0.05% 3,3’-Diaminobenzidine-solution (DAB, Sigma-Aldrich) with 0.01% H_2_O_2_ for 10 min.

To label Anti-Aβ1-16, Anti-GFAP and Anti-Iba1, the dilution medium contained 10% normal serum and 20% Avidin (Vector Labs, Burlingame, USA) diluted in PBS containing 0.2% Triton X-100 (Tx, Sigma-Aldrich, United States). To enhance the staining of GluN1, 1% bovine serum albumin (Sigma-Aldrich, United States) in PBS was used as dilution medium, sections were incubated in the primary AB solutions for 5 days at 4°C and the biotinylated tyramine method (as described by Adams, 1992) was applied.

After mounting on gelatin-coated microscope slides and dehydration, sections were cover-slipped using DPX (Sigma-Aldrich, St. Louis, United States). To exclude unspecific binding of secondary AB and the detection system, negative controls were performed in the same staining protocol by omitting the primary AB. No staining could be observed in these negative controls, indicating that the observed staining was specific.

To assess for changes in protein expression, horizontal sections obtained from −4.6 mm and − 7.34 mm relative to bregma were segregated into regions of interest (ROIs). The ROIs comprised the lateral and medial EC (LEC and MEC), the dentate gyrus (DG) with its granule cell layer (GCL), outer and inner molecular layers (oML and iML), the CA1 and CA3 regions and the subiculum (SB).

#### 2.6.1 Densitometrical analysis

To assess for possible differences in microglia and astrocyte populations, as well as in NMDAR expression between Aβ-treated animals and controls, we performed a macro-densitometrical assessment. The optical density of stained sections, which reflects the level of the respective protein expression, was measured using the software ImageJ 1.48f (US National Institutes of Health, Bethesda, United States).

#### 2.6.2 Astroglia analysis

To investigate possible differences in astrocyte populations between Aβ-treated animals and scrambled peptide-treated controls, we assessed the percentage of area stained positive for GFAP. Analysis was performed using the software ImageJ 1.48f (US National Institutes of Health, Bethesda, United States). Images were converted to 8-bit, then adjusted to an optical density threshold of 170 (on a 0–255 grayscale). Brain regions of interest were selected and the percentage of area above the predetermined threshold was measured.

#### 2.6.3 Assessment of cell viability

To assess cell viability, tissue sections from 4.6 mm and 7.34 mm posterior to Bregma underwent Nissl staining using cresyl violet (CV). For this, sections were mounted on gelatin-coated microscope slides, dehydrated using DPX (Sigma-Aldrich, St. Louis, United States) and cover-slipped. After masked (experimeter-blind) randomization, digital images of the sections were at a 10x magnification and a semi-quantitative assessment was performed, by judging the cell viability of a subregion using a score from 0 (no damage to cells), 1 (only slight chromatin condensation, max. 25% affected), 2 (mediocre chromatin condensation, max. 50% of visible neurons affected), 3 (severe chromatin condensation, max. 75% of visible neurons affected) to 4 (no viable cell visible).

### 2.7 Statistical analysis

Statistical analysis was performed using the STATISTICA software version 14.0.1.25, TIBCO Software Inc., Santa Clara, CA, United States. For *in situ* hybridization data, a one-way analysis of variance (ANOVA) was performed, followed by a Tukey HSD post-hoc test for pairwise comparison (naïve vs. control vs. Aβ). Electrophysiological results were assessed using a multifactorial ANOVA with repeated measures. Behavioral performance and immunohistochemistry results were assessed using the Student’s *t*-test. The level of significance was set at *p* < 0.05. All data were shown as mea*n* ± standard error of mean.

## 3 Results

### 3.1 Progressive impairment of LTP 1 month and 6 months after Aβ-treatment

One month after intracerebral treatment with oligomeric Aβ (1–42) (*n* = 7), we assessed the robustness of LTP in the dentate gyrus of freely behaving rats. After measuring basal synaptic transmission for 1 h, high frequency stimulation (HFS) was applied to the perforant path. This protocol induced long-term potentiation that lasted for over 24 h in control animals (Figure 1A).

By contrast, LTP was significantly reduced in Aβ–treated animals compared to controls (*n* = 7) (Figure 1A): Whereas the initial 15 min of LTP were equivalent in both animal cohorts, deficits became more pronounced from 30 min onwards. Despite the impairments LTP was still present in Aβ-treated animals 24 h after HFS. (ANOVA with repeated measures applied to all time-points recorded after HFS: *F* (1,12) = 7.33, *p* < 0.05).

Examination of LTP in the same animals 6 months after treatment revealed that the LTP impairment had become much more pronounced in Aβ-treated animals (Figure 1B). Now both short-term potentiation and LTP were severely impaired compared to responses evoked in control animals. Evoked potentials had returned to pre-HFS levels by 4 h after stimulation. [ANOVA: *F*(1, 11) = 10.53, *p* < 0.01].

### 3.2 Progressive impairment of object recognition and item-place memory 1 month and 6 months after Aβ-treatment

To test for cognitive impairments, animals engaged in a recognition and a spatial memory task. We observed that 1 month after intracerebral treatment with oligomeric Aβ (1–42), animals (*n* = 15) successfully recognized the new object (*t*-test, *p* < 0.05) with a performance level that was equivalent to controls (*t*-test, *n* = 15, *p* < 0.05) (Figure 1Di). When Aβ–treated animals (*n* = 13) were faced with cognitively more challenging item-place task, memory deficits were evident: only the control animals (*n* = 14) successfully noticed that a familiar object had been moved to a new position (*t*-test, *p* < 0.05) (Figure 1Ci).

By 6 months after treatment, Aβ–treated animals were impaired in both the item-place task (*n* = 12) (Figure 1Cii) and the object recognition task (*n* = 11) (Figure 1Dii). By contrast, control animals exhibited effective memory performance in the item-place task (*t*-test, *n* = 10, p < 0.05) (Figure 1Cii) and the object recognition task (*t*-test, *n* = 14, *p* < 0.05) (Figure 1Dii).

To assess to what extent the memory deficits were caused by changes in information processing in the hippocampus we examined nuclear immediate early gene expression in the hippocampus following item-place learning (Figures 1E–G). The object interactions (not shown) revealed the same outcome as reported in Figure 1Ci [ANOVA *F*(1,16) = 15,93,088, *p* = 0.001; Tukey HSD post-hoc test: control: new vs. old: *p* = 0.0147; Aβ: new vs. old: *p* = 0.1762]. The examination of Homer1a expression was conducted in the suprapyramidal and infrapyramidal blades of the dentate gyrus. The average percentage somatic expression of Homer1a mRNA for both dentate gyrus blades was calculated. The low somatic expression level of Homer1a mRNA in the granule cells of naïve animals (no surgery, no exploration) fits very well with reports from other studies (Vazdarjanova et al. 2002; Hoang et al., 2018). Both groups of animals (*n* = 5 each) that explored the item-place configuration exhibited a significant increase in Homer1a mRNA expression, compared to naïve animals (*n* = 6) (Figure 1F, one-way ANOVA *F*(2,13) = 30.533, *p* = 0.0000, Tukey HSD post-hoc test: control vs. naïve: *p* = 0.0001, Aβ vs. naïve: *p* = 0.0015). This result is in line with observations from other studies reporting that novel spatial learning increases somatic Homer1a expression in the hippocampus (Vazdarjanova et al. 2002; Clifton et al. 2017; Hoang et al., 2018, 2021, 2023). Strikingly, one month after Aβ–treatment, animals (*n* = 5) showed significantly reduced somatic Homer1a expression in the dentate gyrus following the learning task, compared to control animals (*n* = 5) (Figure 1F, Tukey HSD post-hoc test, control vs. Aβ: *p* = 0.0271). This result aligns with the abovementioned behavioral data and indicates that oligomeric Aβ(1–42) causes impairments of experience-dependent information encoding 1 month after intracerebral treatment.

### 3.3 Aβ-treatment results in progressive changes in physiological properties of hippocampal neurons

**Figure 2:**
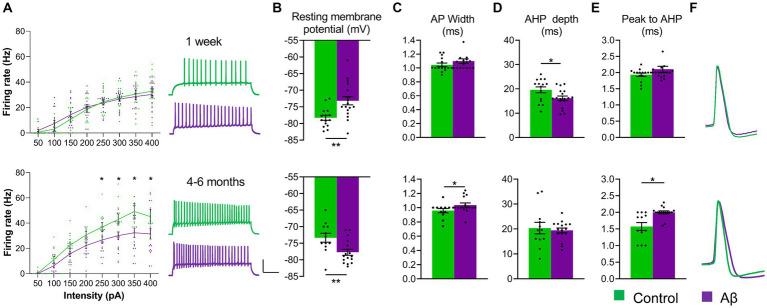
Aβ-treatment results in progressive changes in physiological properties of hippocampal neurones. **(A)** *Left:* The firing frequency of DG granule cells was equivalent in hippocampi from control (scrambled peptide-treated) and Aβ-treated rats 1 week after treatment (top graph) [ANOVA, *F*(1, 248) = 0.283, *p* = 0.595. Aβ: *N* = 8, *n* = 18; control: *N* = 6, *n* = 15], but was significantly reduced in Aβ-treated rats after 4–6 months (bottom graph) [ANOVA, *F*(1, 208) = 32.38, *p* < 0.001, Aβ: *N* = 8, *n* = 16; control: *N* = 7, *n* = 12, Duncan’s test, *, *p* < 0.05]. *Right:* Representative traces showing examples of action potential trains by current intensity 200 pA from Aß-treated (purple) and control (green) tissue recorded 1 week (upper analogs) or 4–6 months after treatment (lower analogs). Vertical scale-bar: 50 mV, horizontal scale-bar: 200 ms. **(B)** Resting membrane potential was significantly less negative 1 week after Aβ-treatment [ANOVA, *F*(1,31) = 9.128, *p* = 0.005] (top chart) (*N* = 8, *n* = 18) compared to controls (*N* = 6, *n* = 15), whereas 4–6 months after Aβ-treatment it became significantly more negative [ANOVA, *F*(1,26) = 8.173, *p* = 0.008] (bottom chart) [Aβ: *N* = 8, *n* = 16; Controls: *N* = 7, *n* = 12]. **(C)** The width of the AP at 20% amplitude was not significantly changed 1 week after Aβ-treatment [ANOVA, F(1,31) = 3.227, *p* = 0.082] (top chart) but was significantly increased after 4–6 months [ANOVA, F(1,26) = 4.806, *p* = 0.037] (bottom chart)(Aβ: *N* = 6, *n* = 16; Controls: *N* = 7, *n* = 12). **(D)** AHP depth was significantly reduced by 1 week after intracerebral Aβ-treatment (ANOVA, F(1,31) = 5.484, *p* = 0.025) (top chart) (Aβ: *N* = 8,*n* = 18; Controls: *N* = 6, *n* = 15), but was not significantly changed 4–6 months after treatment [ANOVA, F(1,26) = 0.184, *p* = 0.670] (bottom chart). **(E)** The latency from AP peak to AHP minimum was unchanged 1 week after Aβ-treatment [ANOVA, F(1,31) = 2.071, *p* = 0.160] (top chart), but 4–6 months after treatment (bottom chart), the latency was significantly increased [ANOVA, F(1,26) = 11.870, *p* = 0.0019] (Aβ:*N* = 6, *n* = 16; Control: *N* = 7, *n* = 12), ANOVA: **, *p* < 0.01; *, *p* < 0.05. **(F)** Representative traces of cell excitability showing analog examples of AP from Aβ-treated (purple) and control (green) tissue recorded 1 week (upper analogs) or 4–6 months after treatment (lower analogs). Data points from individual animals are superimposed upon the graphs and bar charts.

Given that we identified deficits in synaptic plasticity and nuclear expression of Homer1a following Aβ(1–42)-treatment, we explored whether physiological properties of dentate gyrus granule cells were altered. Patch clamp recordings from granule cells revealed no differences in firing frequency when we examined responses one week after Aβ(1–42)-treatment (Aβ: *N* = 8, *n* = 18; control: *N* = 6, *n* = 15, ANOVA: *F*(1, 248) = 0.283, *p* = 0.595) (Figure 2A and S2, top chart), but 4–6 months after treatment, firing frequency was significantly reduced in granule cells from animals that had been treated with Aβ compared to ScrAβ(1–42)- treated controls (Figure 2A and S2, bottom chart) [ANOVA: *F*(1, 208) = 32.38, *p* < 0.001, Aβ: *N* = 8, *n* = 16; control: *N* = 7, *n* = 12, Duncan’s test, *, *p* < 0.05].

Resting membrane potential was significantly more positive in cells from Aβ-treated animals 1 week after treatment (*N* = 8, *n* = 18) (Figure 2B, top chart), compared to controls (*N* = 6, *n* = 15) [ANOVA, *F*(1,31) = 9.128, *p* = 0.005] (top chart), whereas 4–6 months after Aβ–treatment it became significantly more negative (Figure 2B, bottom chart) [ANOVA, *F*(1,26) = 8.173, *p* = 0.008] (bottom chart) (Aβ: *N* = 8, *n* = 16; Controls: *N* = 7, *n* = 12).

The width of the action potential (AP) at 20% of its amplitude was not significantly changed 1 week after Aβ–treatment (Figure 2C, top chart) (ANOVA, F(1,31) = 3.227, *p* = 0.082) but was significantly increased after 4–6 months (Figure 2C, bottom chart) [ANOVA, F(1,26) = 4.806, *p* = 0.037] (bottom chart) (Aβ: *N* = 6, *n* = 16; Controls: *N* = 7, *n* = 12).

Afterhyperpolarization (AHP) depth was significantly reduced in cells from Aβ-treated animals 1 week after treatment (Figure 2D, top chart) [ANOVA, F(1,31) = 5.484, *p* = 0.025] (Aβ: *N* = 8, *n* = 18; Controls: *N* = 6, *n* = 15), but not significantly changed 4–6 months after treatment [ANOVA, F(1,26) = 0.184, *p* = 0.670) (Figure 2D, bottom chart].

The latency from AP peak to AHP minimum was unchanged 1 week after Aβ-treatment (Figure 2E, top chart) [ANOVA, F(1,31) = 2.071, *p* = 0.160] (top chart), but 4–6 months after treatment (bottom chart), the latency was significantly increased (Figure 2E, bottom chart). [ANOVA, F(1,26) = 11.870, *p* = 0.0019 (Aβ: *N* = 6, *n* = 16; Control: *N* = 7, *n* = 12), ANOVA: **, *p* < 0.01; *, *p* < 0.05].

Taken together, these data suggest that early, but subtle increases in membrane excitability are succeeded months after treatment by a reduction in neural responsiveness.

### 3.4 Intracerebral inoculation with Aβ(1–42) does not trigger the propagation of amyloid plaques and does not affect cell viability in the hippocampus by 6 months after treatment

**Figure 3:**
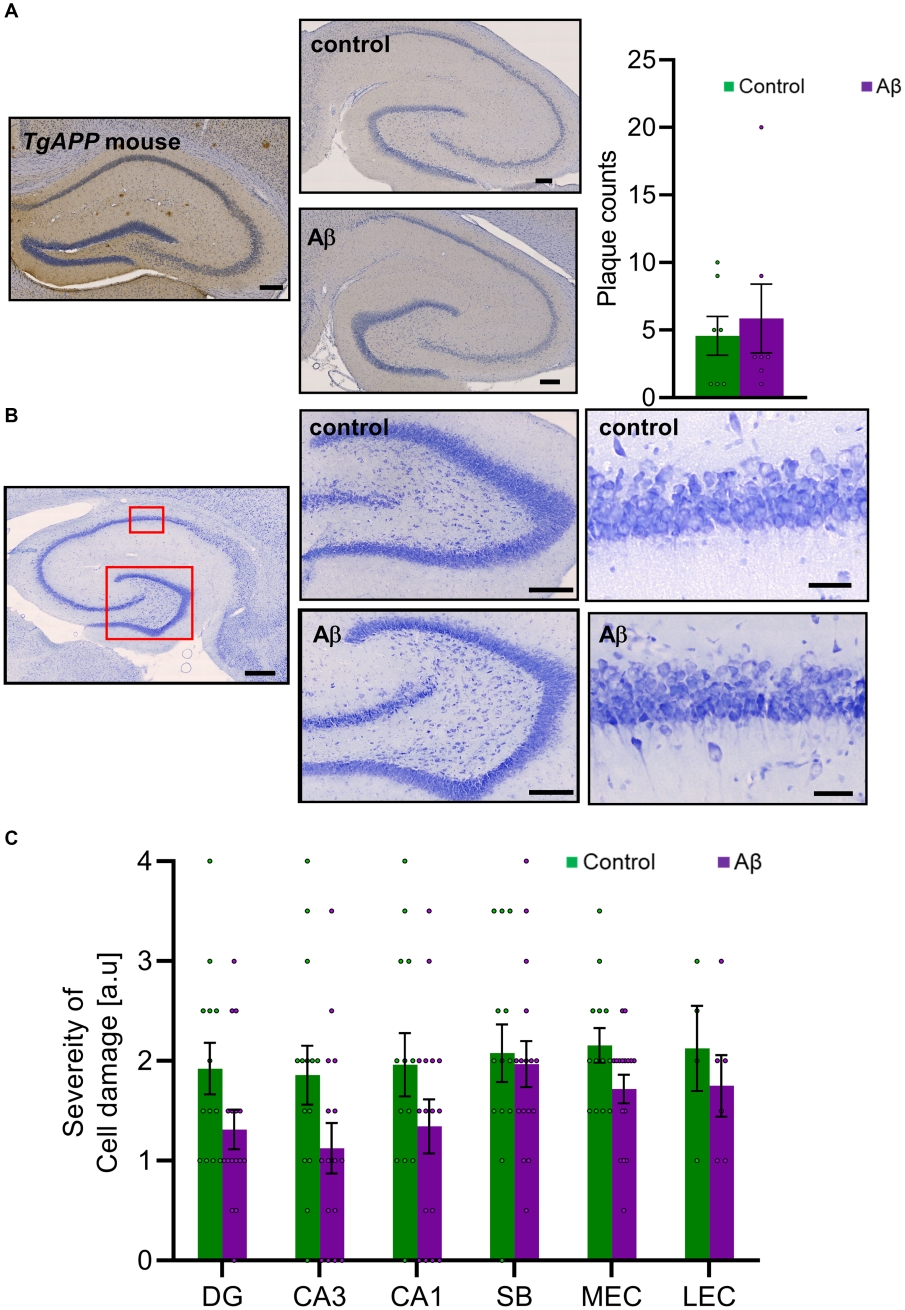
Aβ-treatment neither changes amyloid plaque levels nor affects cell viability, **(A)** *Left:* DAB-labeled immunohistochemical photomicrographs show examples of Aβ-plaques in hippocampus of a transgenic mouse that express amyloid precursor leading to plaque development (Tg*APP*, 9 months old, as a positive control) and in an Aβ-treated rat (6 months old) compared to control (6 M). Scale bar: 200 μm. *Right:* Bar chart shows plaque counts in rat brains following Aβ-treatment compared to control. DAB-labeled immunohistochemistry revealed no significant changes of amyloid plaque loads 6 months after Aβ-treatment (Aβ: *n* = 8; Control: *n* = 7; *t*-test: T (13) = 0.242, *p* = 0.812). **(B)** Nissl-stained photomicrographs show examples of cells in hippocampus (scale bar: 500 μm), in the dentate gyrus and CA1 region (scale bar: 50 μm). **(C)** Cell viability was not significantly changed between Aβ (*n* = 8) and control (*n* = 15) groups 6 months after the initial treatment based on a semi-quantitative assessment of Nissl-stained sections in CA1, the DG, the SB, the lEC, or the mEC. *t*-test: *p* > 0.05. DAB: 3,3’-Diaminobenzidine-solution; DG: dentate gyrus; DG GCL: granule cell layer of the dentate gyrus; DG oML: outer molecular layer of the dentate gyrus; DG iML; inner molecular layer of the dentate gyrus; SB: subiculum; mEC: medial entorhinal cortex; lEC: lateral entorhinal cortex. Data points from individual animals are superimposed upon the bar charts.

In the current study, immunohistochemistry assessments did not detect an increase in the number of Aβ-plaques 6 months after Aβ-treatment (*n* = 8) compared to control animals (*n* = 7) (Figure 3A) [*t*-test: T(13) = 0.242, *p* = 0.812]. To verify that this result was not due to problems with the antibody used, we assessed for plaque expression in a transgenic mouse model of AD (*TgAPP,* 9 month old mice) and were able to detect plaques (Figure 3A).

We scrutinized the hippocampus, the lateral and medial entorhinal cortex, and the subiculum and discovered that in 6 month old *control* animals a small number of plaques are intrinsically present (Figure 3A). Levels in Aβ-treated animals did not differ from levels detected in controls, however.

We then assessed Nissl stained brain slices to examine for cell damage in the hippocampal CA1 region, the dentate gyrus, the subiculum, the lateral entorhinal cortex, or the medial entorhinal cortex (Figures 3B,C). We found no evidence of increased cytoxicity in Aβ-animals (*n* = 8), 6 months after treatment, compared to controls (*n* = 7) (Table 1).

**Table 1:** Statistical analysis of cell viability in oligomeric Aβ (1–42)-treated (*n* = 8) and control animals (*n* = 7) 6 months after treatment.

Brain region	Test group	Cell viability score	*t*-test	*p*-value
Dentate gyrus	Aβ	1.31 ± 0.20	T (27) = −1.907	*p* = 0.067
	Control	1.92 ± 0.26		
CA3	Aβ	1.13 ± 0.25	T (27) = −1.804	p = 0.082
	Control	1.85 ± 0.31		
CA1	Aβ	1.34 ± 0.27	T (27) = −1.483	*p* = 0.150
	Control	1.96 ± 0.32		
Subiculum	Aβ	1.97 ± 0.23	T (27) = −0.297	*p* = 0.769
	Control	2.08 ± 0.28		
Lateral entorhinal cortex	Aβ	1.75 ± 0.30	T (8) = −0.730	*p* = 0.486
	Control	2.13 ± 0.43		
Medial entorhinal cortex	Aβ	1.72 ± 0.14	T (27) = −1.946	*p* = 0.062
	Control	2.15 ± 0.17		

### 3.5 Aβ–treatment results in transient activation of microglia and chronic loss of astrocytes in the hippocampus

**Figure 4:**
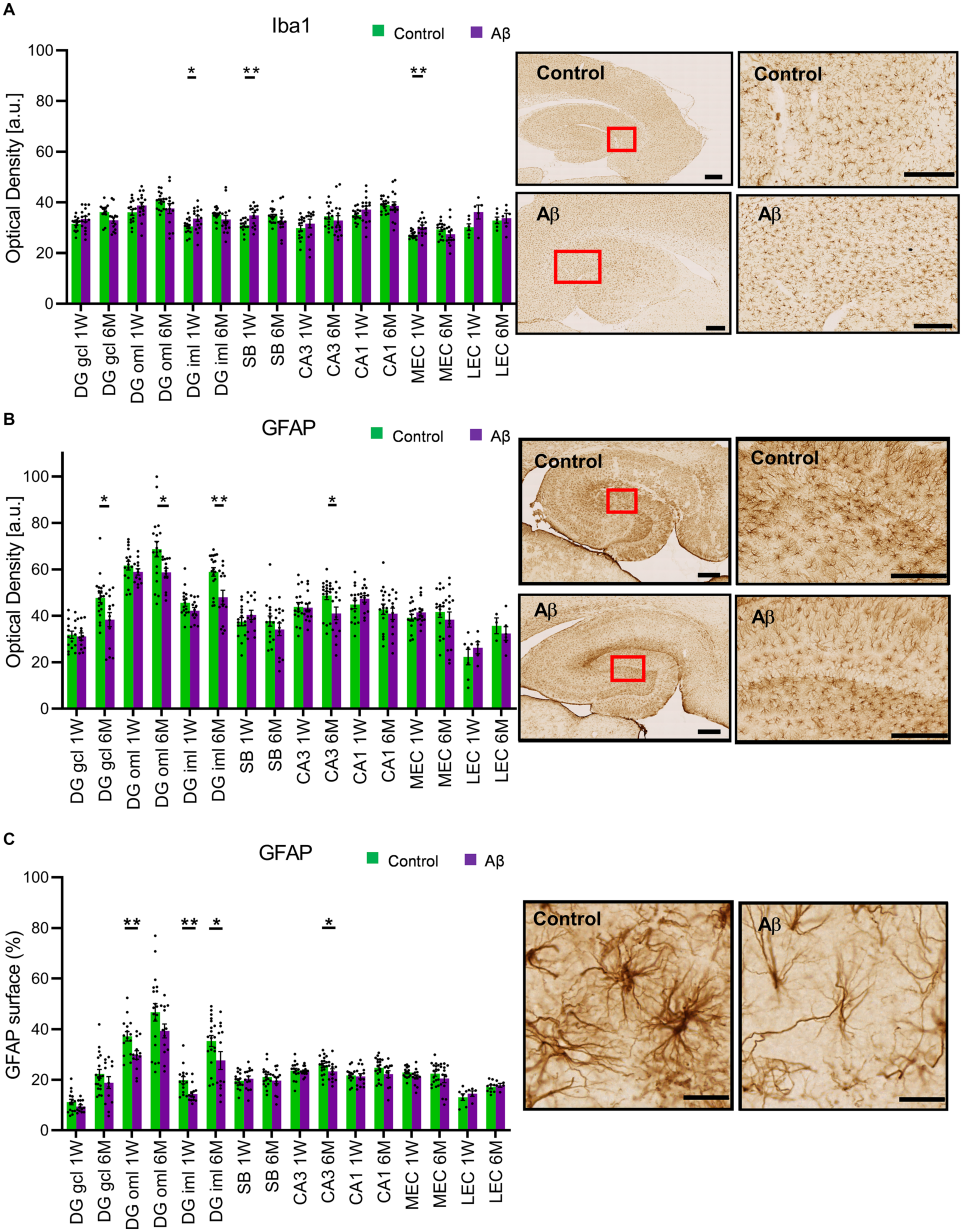
Increase in Iba1 expression occurs after Aβ-treatment, whereas GFAP expression decreases in a time-dependent manner. **(A)** *Left:* Iba1 expression was significantly increased 1 week (1 W) but not 6 months (6 M) after Aβ-treatment, in the DG iML, the SB and the mEC. *Right:* Photomicrographs show examples of Iba1 expression in hippocampus (left, scale bar: 500 μm) and subiculum (right, scale bar: 50 μm) 1 W after Aβ-treatment. *t*-test **p < 0.01, *p < 0.05. **(B)** *Left:* Densitometric analysis of GFAP expression 1 week (1 W) or 6 months (6 M) after Aβ-treatment. Significant decreases in expression are evident in the DG GCL, the DG oML and the DG iML, as well as in the CA3 region 6 M, but not 1 W after Aβ-treatment. The other areas assessed were unaffected. *Right:* Photomicrographs show examples of GFAP expression in hippocampus (left, scale bar: 500 μm) and DG (right, scale bar: 50 μm): 6 M control vs. 6 M Aβ. *t*-test **p < 0.01, *p < 0.05. **(C)** *Left:* Area analysis of GFAP expression 1 week (1 W) or 6 months (6 M) after Aβ-treatment. Significant decreases in expression are evident in the DG oML and the DG iML 1 W after Aβ-treatment. DG iML and CA3 region also exhibit significant decreases in GFAP expression 6 M after Aβ-treatment. The other areas assessed were unaffected. *Right:* Photomicrographs show reduction in arborisation as well as surface area of GFAP positive cells in iML/oML of DG in a Aβ-treated animal (6 M) compared to control (6 M). Scale bar: 20 μm. *t*-test **p < 0.01, *p < 0.05. DG: dentate gyrus; DG GCL: granule cell layer of the dentate gyrus; DG oML: outer molecular layer of the dentate gyrus; DG iML; inner molecular layer of the dentate gyrus; GFAP: fibrillary acidic protein; Iba1: ionized calcium-binding adapter molecule 1; SB: subiculum; mEC: medial entorhinal cortex; lEC: lateral entorhinal cortex. Data points from individual animals are superimposed upon the bar charts.

A role for microglia in AD progression has been proposed (Edwards, 2019). Here, we used immunohistochemical analysis of Iba1 expression to assess to what extent microglia are activated in the hippocampal CA1 region, the dentate gyrus, the subiculum, the lateral entorhinal cortex and the medial entorhinal cortex at early (1 week) or late (6 month) latencies after Aβ-treatment (Figure 4A). We found that Iba1 expression was increased in the inner molecular layer of the dentate gyrus, the subiculum, the medial entorhinal cortex 1 week after Aβ-treatment (*n* = 4), compared to controls (*n* = 4) (Table 2). By contrast, 6 months after treatment Iba1 expression levels were equivalent in Aβ-treated (*n* = 4) and control animals (*n* = 4) (Table 3).

**Table 2:** Statistical analysis of Iba1 expression in Aβ–treated (*n* = 4) and control animals (*n* = 4) 1 week after treatment.

Brain region	Test group	Densitometric value	*t*-test	*p*-value
DG GCL	Aβ	33.46 ± 1.06	T (29) = −1.479	*p* = 0.150
	Control	31.56 ± 0.68		
DG oML	Aβ	38.75 ± 1.28	T (29) = −1.537	*p* = 0.135
	Control	36.11 ± 1.13		
DG iML	Aβ	33.59 ± 1.13	T (29) = −2.222	*p* = 0.034*
	Control	30.51 ± 0.77		
CA3	Aβ	31.66 ± 1.70	T (27) = −0.888	*p* = 0.382
	Control	29.89 ± 1.09		
CA1	Aβ	37.19 ± 1.33	T (29) = −1.417	*p* = 0.167
	Control	35.04 ± 0.66		
Subiculum	Aβ	34.89 ± 1.10	T (26) = −3.093	*p* = 0.004 *
	Control	30.92 ± 0.65		
Lateral entorhinal cortex	Aβ	36.18 ± 2.74	T (9) = −2.069	*p* = 0.068
	Control	30.19 ± 1.36		
Medial entorhinal cortex	Aβ	30.29 ± 0.84	T (26) = −3.112	*p* = 0.004 *
	Control	27.21 ± 0.40		

DG GCL, dentate gyrus granule cell layer; DG oML, dentate gyrus outer molecular layer; DG iML, dentate gyrus innermolecular layer.

**Table 3:** Statistical analysis of Iba1 expression in Aβ-treated (*n* = 4) and control animals (*n* = 4) 6 months after treatment.

Brain region	Test group	Densitometric value	*t*-test	*p*-value
DG GCL	Aβ	33.07 ± 1.10	T (25) = −2.052	*p* = 0.051
	Control	36.23 ± 1.03		
DG oML	Aβ	38.14 ± 1.89	T (29) = −1.212	*p* = 0.235
	Control	40.59 ± 0.82		
DG iML	Aβ	33.46 ± 1.53	T (29) = −1.081	*p* = 0.288
	Control	35.20 ± 0.59		
CA3	Aβ	33.08 ± 1.76	T (28) = −0.686	*p* = 0.498
	Control	34.51 ± 1.10		
CA1	Aβ	38.93 ± 1.49	T (29) = −0.044	*p* = 0.965
	Control	39.01 ± 0.65		
Subiculum	Aβ	33.10 ± 1.32	T (29) = −1.037	*p* = 0.309
	Control	34.62 ± 0.68		
Lateral entorhinal cortex	Aβ	33.78 ± 1.89	T (10) = 0.377	*p* = 0.714
	Control	32.92 ± 1.33		
Medial entorhinal cortex	Aβ	27.67 ± 1.23	T (27) = −1.165	*p* = 0.254
	Control	29.34 ± 0.77		

DG GCL, dentate gyrus granule cell layer; DG oML, dentate gyrus outer molecular layer; DG iML, dentate gyrus innermolecular layer.

To examine to what extent astrocytes might be affected by Aβ-treatment we assessed GFAP levels 1 week and 6 months after treatment (Figures 4B,C) using densitometrical analysis (Tables 4, 5) and an analysis of the percentage of area covered by GFAP positive astrocytes (Tables 6, 7). GFAP is an astroglia cytoskeleton-specific protein which is upregulated in response to reactive astrogliosis (Jurga et al., 2021). We detected reconstruction in the morphology of GFAP-positive cells in Aβ-treated animals, compared to control animals (Figure 4C, photomicrographs). The astrocytes in Aβ-treated animals exhibited a reduction in their complexity (i.e., in arborisation and surface area) compared to control animals. Densitometrical analysis revealed no changes in GFAP expression levels in Aβ-treated (*n* = 4) and control animals (*n* = 4) 1 week after treatment (Table 4), whereas the area analysis revealed significant reductions of astrocyte density (Table 5). Six months after treatment, Aβ-treated animals (*n* = 4) exhibited a significant reduction of GFAP expression in the granule cell, the outer and inner molecular layer of the dentate gyrus and the CA3 region compared to controls (*n* = 4) in the densitometrical analysis (Table 6), and in the outer molecular layer of the dentate gyrus and the CA3 region in the area analysis (Table 7). Our observations are in line with findings by others, that suggest that a reduction in GFAP positive astrocytes is correlated with changes in astroglial morphology, which occurs during pathological states in the brain (Senitz et al., 1995; Chvátal et al., 2007a,b).

**Table 4:** Statistical analysis of GFAP expression in Aβ–treated (*n* = 4) and control animals (*n* = 4) 1 week after treatment.

Brain region	Test group	Densitometric value	*t*-test	*p*-value
DG GCL	Aβ	31.07 ± 1.55	T (27) = 0.356	*p* = 0.725
	Control	31.87 ± 1.56		
DG oML	Aβ	58.89 ± 1.36	T (27) = 1.125	*p* = 0.271
	Control	61.63 ± 1.89		
DG iML	Aβ	42.24 ± 1.36	T (27) = 1.644	*p* = 0.112
	Control	45.64 ± 1.49		
CA3	Aβ	43.46 ± 1.76	T (28) = 0.164	*p* = 0.871
	Control	43.89 ± 1.82		
CA1	Aβ	47.30 ± 1.45	T (28) = −1.014	*p* = 0.319
	Control	44.86 ± 1.86		
Subiculum	Aβ	40.34 ± 2.02	T (26) = −1.065	*p* = 0.297
	Control	37.48 ± 1.79		
Lateral entorhinal cortex	Aβ	26.27 ± 2.56	T (10) = −0.881	*p* = 0.399
	Control	22.28 ± 3.34		
Medial entorhinal cortex	Aβ	41.48 ± 1.44	T (27) = −1.114	*p* = 0.275
	Control	39.18 ± 1.48		

DG GCL, dentate gyrus granule cell layer; DG oML, dentate gyrus outer molecular layer; DG iML, dentate gyrus innermolecular layer.

**Table 5:** Statistical analysis of GFAP positive area in regions of interest in Aβ–treated (*n* = 4) and control animals (*n* = 4) 1 week after treatment.

Brain region	Test group	% GFAP pos. Area	*t*-test	*p*-value
DG GCL	Aβ	9.46 ± 0.62	T (26) = 1.326	*p* = 0.197
	Control	11.19 ± 1.09		
DG oML	Aβ	29.77 ± 1.69	T (26) = 2.887	p = 0.008*
	Control	37.20 ± 1.89		
DG iML	Aβ	14.33 ± 0.92	T (26) = 3.189	p = 0.004*
	Control	19.86 ± 1.40		
CA3	Aβ	23.35 ± 0.47	T (27) = 0.230	*p* = 0.820
	Control	23.59 ± 0.92		
CA1	Aβ	21.36 ± 1.02	T (26) = 0.301	*p* = 0.766
	Control	21.75 ± 0.80		
Subiculum	Aβ	20.40 ± 1.13	T (27) = −0.559	*p* = 0.581
	Control	19.62 ± 0.84		
Lateral entorhinal cortex	Aβ	14.52 ± 1.07	T (10) = −0.775	*p* = 0.456
	Control	13.14 ± 1.29		
Medial entorhinal cortex	Aβ	21.14 ± 0.82	T (27) = 1.830	*p* = 0.078
	Control	22.91 ± 0.54		

DG GCL, dentate gyrus granule cell layer; DG oML, dentate gyrus outer molecular layer; DG iML, dentate gyrus innermolecular layer.

**Table 6:** Statistical analysis of GFAP expression in Aβ-treated (*n* = 4) and control animals (*n* = 4) 6 months after treatment.

Brain region	Test group	Densitometric value	*t*-test	*p*-value
DG GCL	Aβ	38.33 ± 3.04	T (31) = 2.644	*p* = 0.013*
	Control	47.75 ± 2.08		
DG oML	Aβ	58.75 ± 1.70	T (31) = 2.406	*p* = 0.029*
	Control	68.68 ± 3.30		
DG iML	Aβ	47.96 ± 3.04	T (31) = 3.236	*p* = 0.002*
	Control	57.76 ± 2.86		
CA3	Aβ	41.06 ± 2.70	T (31) = 2.484	*p* = 0.019*
	Control	48.70 ± 1.73		
CA1	Aβ	42.81 ± 2.24	T (28) = 0.569	*p* = 0.573
	Control	40.90 ± 2.45		
Subiculum	Aβ	34.18 ± 2.71	T (29) = 1.022	*p* = 0.315
	Control	37.73 ± 2.21		
Lateral entorhinal cortex	Aβ	32.44 ± 2.92	T (7) = 0.804	*p* = 0.516
	Control	35.74 ± 3.39		
Medial entorhinal cortex	Aβ	38.38 ± 3.24	T (28) = 0.832	*p* = 0.412
	Control	41.63 ± 2.30		

DG GCL, dentate gyrus granule cell layer; DG oML, dentate gyrus outer molecular layer; DG iML, dentate gyrus inner molecular layer.

**Table 7:** Statistical analysis of GFAP positive area in regions of interest in Aβ–treated (*n* = 4) and control animals (*n* = 4) 6 months after treatment.

Brain region	Test group	% GFAP pos. Area	*t*-test	*p*-value
DG GCL	Aβ	18.48 ± 2.23	T (31) = 1.508	*p* = 0.142
	Control	22.31 ± 1.74		
DG oML	Aβ	37.65 ± 2.63	T (31) = 1.763	*p* = 0.088
	Control	46.68 ± 3.44		
DG iML	Aβ	26.11 ± 3.24	T (31) = 2.237	*p* = 0.033*
	Control	35.34 ± 2.17		
CA3	Aβ	23.05 ± 1.02	T (31) = 2.131	*p* = 0.041*
	Control	25.71 ± 0.82		
CA1	Aβ	21.83 ± 1.19	T (29) = 1.889	*p* = 0.069
	Control	24.36 ± 1.00		
Subiculum	Aβ	19.76 ± 1.22	T (29) = 0.887	p = 0.382
	Control	21.00 ± 0.90		
Lateral entorhinal cortex	Aβ	17.78 ± 0.63	T (12) = −0.576	*p* = 0.575
	Control	17.16 ± 0.76		
Medial entorhinal cortex	Aβ	19.81 ± 1.62	T (29) = 1.437	*p* = 0.161
	Control	22.31 ± 0.91		

DG GCL, dentate gyrus granule cell layer; DG oML, dentate gyrus outer molecular layer; DG iML, dentate gyrus innermolecular layer.

Taken together, these data suggest that an early and transient activation of microglia is followed by a change in morphology of astrocytes following Aβ-treatment.

### 3.6 Aβ-treatment results in changes in the expression of NMDAR

**Figure 5:**
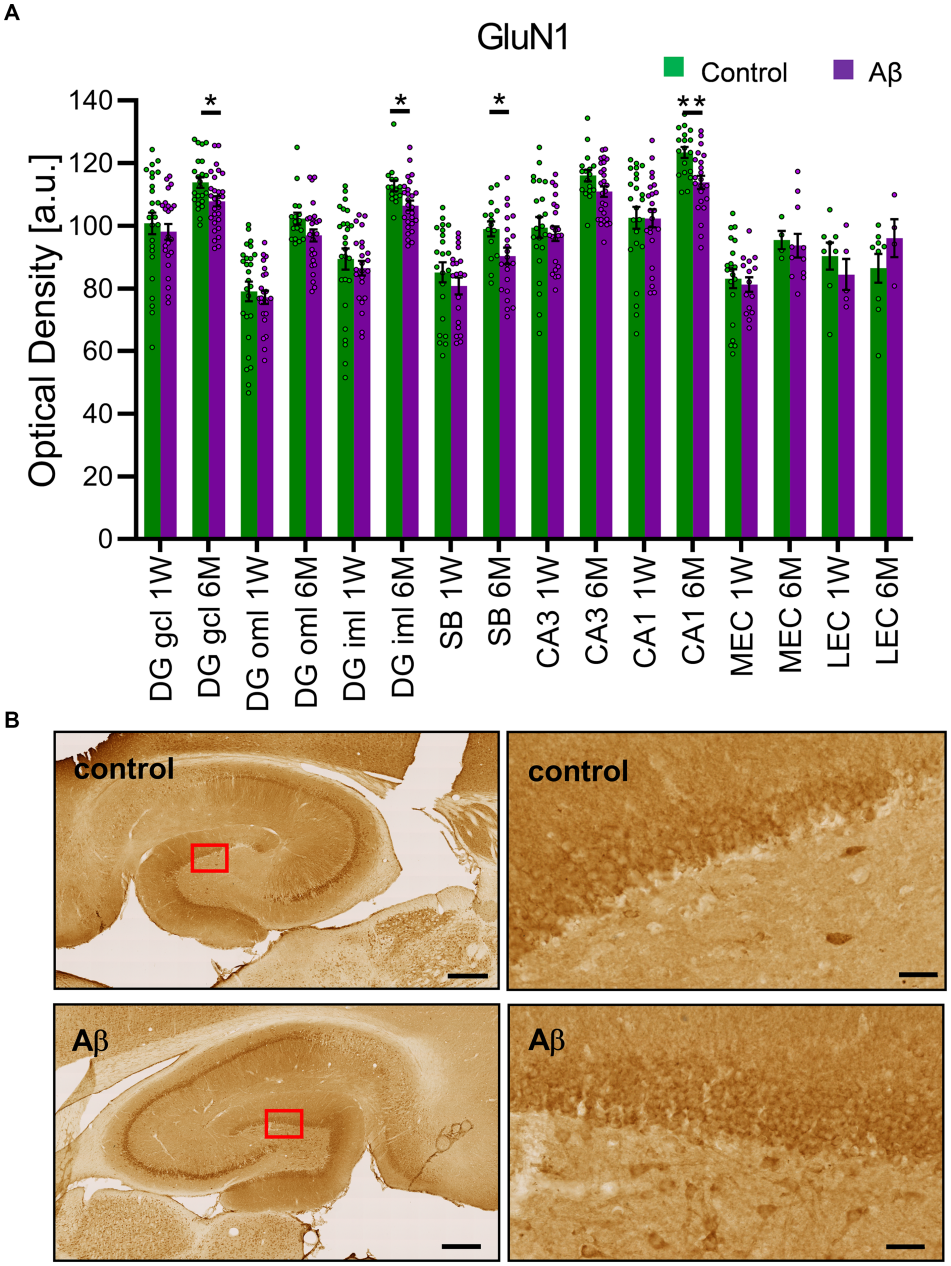
Aβ-treatment leads to significant reduction of GluN1 expression in the hippocampus. **(A)** GluN1 expression in the DG, the SB, the CA1, the CA3, mEC and lEC 1 week or 6 months after control (*n* = 8) and Aβ-treatment (*n* = 7). GluN1 expression was significantly decreased 6 months but not 1 week after treatment in the DG GCL, DG iML, the CA1 region and the SB. *t*-test **p < 0.01, *p < 0.05. **(B)** Photomicrographs show examples of GluN1 expression in the hippocampus (left, scale bar: 500 μm) and dentate gyrus granule cell layer (right, scale bar: 50 μm) in 6 M control and 6 M Aβ. DG: dentate gyrus; DG GCL: granule cell layer of the dentate gyrus; DG oML: outer molecular layer of the dentate gyrus; DG iML; inner molecular layer of the dentate gyrus; SB: subiculum; mEC: medial entorhinal cortex; lEC: lateral entorhinal cortex. Data points from individual animals are superimposed upon the bar charts.

Changes in NMDAR function have been proposed to contribute to the impairments of LTP seen in various animal models of AD (Rammes et al., 2018; Yang et al., 2018). Here, we investigated the expression of the GluN1 subunit of the NMDAR in the CA1 region, the dentate gyrus, the subiculum, the lateral entorhinal cortex and the medial entorhinal cortex (Figure 5). No changes in receptor expression were detected 1 week after treatment when effects were compared in Aβ-treated (*n* = 8) and control animals (*n* = 7) (Table 8). However, 6 months after treatment we detected a significant decrease in GluN1 expression in the granule cell and the inner molecular layers of the dentate gyrus, as well as in the subiculum and the CA1 regions of Aβ-treated animals (*n* = 8), compared to controls (*n* = 7) (Table 9).

**Table 8:** Statistical analysis of GluN1 expression in Aβ-treated (*n* = 8) and control animals (*n* = 7) 1 week after treatment.

Brain region	Test group	Densitometric value	*t*-test	*p*-value
DG GCL	Aβ	98.08 ± 2.53	T(46) = 0.622	*p* = 0.537
	Control	100.76 ± 3.40		
DG oML	Aβ	77.18 ± 2.05	T(47) = 0.476	*p* = 0.636
	Control	79.01 ± 3.12		
DG iML	Aβ	86.47 ± 2.29	T(47) = 0.691	*p* = 0.493
	Control	89.39 ± 3.42		
CA3	Aβ	97.43 ± 2.30	T(42) = 0.498	*p* = 0.621
	Control	99.44 ± 3.32		
CA1	Aβ	102.33 ± 2.86	T(44) = 0.045	p = 0.965
	Control	102.53 ± 3.47		
Subiculum	Aβ	80.79 ± 2.67	T(40) = 1.020	*p* = 0.314
	Control	85.10 ± 3.22		
Lateral entorhinal cortex	Aβ	84.41 ± 4.98	T(11) = 0.877	*p* = 0.399
	Control	90.31 ± 4.30		
Medial entorhinal cortex	Aβ	81.24 ± 2.34	T(33) = 0.453	*p* = 0.654
	Control	83.09 ± 3.06		

DG GCL, dentate gyrus granule cell layer; DG oML, dentate gyrus outer molecular layer; DG iML: dentate gyrus inner molecular layer.

**Table 9:** Statistical analysis of GluN1 expression in Aβ-treated (*n* = 8) and control animals (*n* = 7) 6 months after treatment.

Brain region	Test group	Densitometric value	*t*-test	*p*-value
DG GCL	Aβ	107.83 ± 1.65	T (50) = −2.517	*p* = 0.015*
	Control	113.79 ± 1.67		
DG oML	Aβ	96.92 ± 1.93	T (42) = −1.864	*p* = 0.069
	Control	102.28 ± 1.91		
DG iML	Aβ	106.45 ± 1.54	T (40) = −2.599	*p* = 0.013*
	Control	112.76 ± 1.70		
CA3	Aβ	110.90 ± 1.70	T (42) = −1.960	*p* = 0.057
	Control	116.01 ± 1.86		
CA1	Aβ	113.89 ± 2.11	T (38) = −3.351	*p* = 0.001*
	Control	123.35 ± 1.75		
Subiculum	Aβ	90.38 ± 2.62	T (22) = −2.292	*p* = 0.028*
	Control	98.97 ± 2.41		
Lateral entorhinal cortex	Aβ	96.32 ± 4.42	T (14) = −1.514	*p* = 0.152
	Control	86.46 ± 4.60		
Medial entorhinal cortex	Aβ	93.66 ± 3.77	T (13) = −0.267	*p* = 0.793
	Control	95.43 ± 2.93		

DG GCL, dentate gyrus granule cell layer; DG oML, dentate gyrus outer molecular layer; DG iML, dentate gyrus inner molecular layer.

## 4 Discussion

In this study we show that a single, once-off, intracerebral inoculation of *healthy* non-transgenic adult rats with oligomeric Aβ(1–42) sets a progressive and pathological cascade in motion, from which the hippocampus does not recover (summarized in Supplementary file S1). Initial deficits in LTP become progressively worse over the 6-month observation period after Aβ–treatment. Although animals are initially able to master a relatively simple cognitive task, they fail in acquiring item-place memory. Six months after treatment they fail in a simple object recognition task. These deficits are accompanied by cumulative changes in active neuronal properties, but changes in cell viability and accumulation of amyloid plaques do not occur. Rather, we detected evidence of an early activation of microglia, followed by a reduction in astroglial complexity suggesting that immune and housekeeping strategies of the brain are engaged, or perhaps compromised, by the presence of oligomeric Aβ(1–42). The particular novelty of this finding lies in the fact that animals were healthy and did not have a transgenically manipulated predisposition to Aβ or AD-pathology. Our findings suggest that a single perturbation of Aβ housekeeping in the healthy brain can lead to a potent and persistent debilitation of hippocampal function.

We treated adult rats intracerebroventricularly *only once* with oligomeric Aβ(1–42), using a dose that results in acute impairment of LTP in behaving rats (Kalweit et al., 2015). One month after treatment, LTP was significantly reduced in the dentate gyrus (DG) *in vivo*. Animals were also impaired in a spatial recognition task. Furthermore, following a spatial learning task that typically facilitates synaptic plasticity in DG (Kemp and Manahan-Vaughan, 2004), somatic expression of the immediate early gene, Homer1a, was also reduced 1 month after treatment, suggesting that information processing and encoding in the hippocampus is compromised by oligomeric Aβ(1–42). This early loss of the ability of the hippocampus to encode and retain synaptic and spatial memory may correspond to early stages of sporadic AD, where mild memory impairments occur that can be circumvented by boosting attention levels either behaviorally or pharmacologically (Cavallo et al., 2016; Huntley et al., 2016). In line with this interpretation, we observed that increasing the attentional demand of the spatial learning task by introducing novel objects enabled a learning performance in Aβ-treated rats that was equivalent to controls 1 month after treatment. To clarify if the loss in hippocampal plasticity and spatial learning impairments were transient or long-lasting, we examined LTP 6 months after treatment and found that LTP was still impaired. Furthermore, at this time-point after Aβ-treatment, not only spatial recognition memory, but now additionally object recognition memory was impaired. This suggests that Aβ treatment triggered persistent and progressive changes in hippocampal function.

Aberrant patterns of neuronal excitability have been reported in both animal models and human AD patients (Yun et al., 2006; D'Amelio and Rossini, 2012). We therefore examined if changes in neuronal excitability underlie the long-term hippocampal impairments that we observed in the present study. Whole-cell patch clamp recordings of DG granule cells revealed that 1 week after Aβ-treatment neuronal excitability was enhanced, as indicated by a less negative resting membrane potential and reduced afterhyperpolarisation (AHP) depth, whereas no significant changes in firing frequency and input resistance was found. By contrast, 4–6 months after Aβ-treatment, neuronal firing rates were significantly reduced, indicating that reduced neuronal excitability succeeded the initial increases observed 1 week after treatment. In line with this, the resting membrane potential became more negative, the action potential (AP) exhibited an increased width and the latency from AP peak to AHP was prolonged. These data suggest that an altered excitation-inhibition contributes to the changed hippocampal function we observed following Aβ-treatment. Although hyperexcitability and epileptic activity are often observed in old, plaque-burdened transgenic mice that overexpress Aβ (Palop and Mucke, 2010; Gurevicius et al., 2013; Kellner et al., 2014), in young, pre-plaque transgenic mice, the pattern of change is not yet clear: both hyper- and hypoexcitability have been reported (Palop et al., 2007; Minkeviciene et al., 2009). Intriguingly, aged-related reductions in intrinsic excitability have also been observed (Landfield and Pitler, 1984; Disterhoft and Oh, 2007; Wang et al., 2011; Huntley et al., 2016), suggesting that Aβ-treatment may have accelerated intrinsic age-related changes in the hippocampus.

A prion-like mechanism supports Aβ-deposition in the brains of genetically modified mice (Goedert, 2015; Jucker and Walker, 2018) and it was reported that human transmission of amyloid can occur (Jaunmuktane et al., 2015; Banerjee et al., 2024). In the late stages of AD, plaques are often evident. However, Aβ oligomers, but not fibrils (a primary component of plaques), disrupt hippocampal synaptic plasticity and cognition (Walsh et al., 2002) and Aβ-seeding activity can develop in the absence of plaques (Rother et al., 2022). We explored whether Aβ plaques developed as a result of Aβ-treatment and found that 6 months after treatment, the number of amyloid plaques was not significantly changed in the brain. This suggests that the changes in hippocampal function that we observed were not mediated by the presence of plaques. At the same time, cell viability remained comparable between treatment groups, indicating that Aβ-treatment did not exert its effects via cytotoxicity, or widespread cell loss. Thus, the time-points after Aβ-treatment that we examined may correspond to the early pathogenesis of amyloidosis.

We then explored which molecular mechanisms could underlie the effects we observed., and first scrutinized whether neuroinflammatory processes were triggered by Aβ-treatment. We found that Iba1, that is expressed in microglia and is upregulated when they are activated (Ito et al., 1998), exhibited increased expression 1 week after Aβ-treatment in the dentate gyrus, subiculum, as well as in the medial entorhinal cortex. Six months after Aβ-treatment changes in Iba1 expression were no longer evident. Instead, at this time-point the expression of glia fibrillary acidic protein (GFAP), an astrocyte biomarker (Yang and Wang, 2015), was significantly reduced in the dentate gyrus and CA3 regions, suggesting that an initial microglia activation was succeeded by a loss of function and reactivity of astrocytes, a process that may contribute to brain aging and to neurodegeneration in AD (Birch, 2014; De Strooper and Karran, 2016). More recently it has been proposed that plasma GFAP is elevated in patients exhibiting mild cognitive impairment coupled with amyloidosis, and that GFAP-astrocyte labeling correlates specifically with accumulating Aβ in a transgenic mouse model of amyloidosis (De Bastiani et al., 2023). Furthermore, reactive astrogliosis in patients may promote the early pathogenesis of AD (Pelkmans et al., 2024). A corollary of these findings is that that animal model we used for the present study may allow the scrutiny of the pathophysiology of the early pathogenesis of amyloidosis in AD.

Activation of NMDAR is a key molecular step in the induction of LTP and the enablement of hippocampus-dependent memory (Paoletti et al., 2013). Although the NMDAR exists in many subunit variants, the GluN1 subunit is contained in every NMDAR (Hansen et al., 2018) and thus serves as a useful biomarker of NMDAR expression. We observed a significant reduction in hippocampal and subicular expression of the GluN1 subunit 6 months after Aβ-treatment. Many genetic animal models of AD report that increased neuronal excitability or excitotoxicity coexist with an increased burden of amyloid plaques (Davis et al., 2014), but plaques first emerge at 6 months of age or later in mice that overexpress Aβ. Our data suggest that in earlier stages of the progression of Aβ-mediated changes in AD, and in particular in rodents that were *not* genetically modified to overexpress Aβ, NMDAR hypofunction coupled with a changed excitatory: inhibitory balance and alterations in astrocyte function, may underlie early hippocampal deficits in AD. These findings align with reports by others of the impact of oligomeric Aβ in rodents, or amyloidosis in patients (Foster et al., 2017; Li and Selkoe, 2020), and reinforce our abovementioned proposal that intracerebral inoculation of the brains of healthy *non-transgenic* rodents with oligomeric Aβ(1–42) may serve as a useful model for the study of the mechanisms underlying the early pathogenesis of AD driven by amyloidosis.

The progress of hippocampal changes following Aβ-treatment was insidious and subtle. Given that we found no evidence of toxicity *per se* and no plaques, it is tempting to speculate that the single Aβ-inoculation led to propagation of Aβ(1–42). Amyloidosis has been proposed to be a determinant of early cognitive decline in AD, leading to the hypothesis that it is an initiator of AD-pathophysiology (Wang et al., 2015). Inoculation of brain tissue with Aβ, referred to as ‘seeding’ leads to amyloidosis in transgenic animal modes of AD, in a process akin to prion protein propagation (Jucker and Walker, 2013, 2018) and Aβ-oligomers accelerate Aβ-seeding (Katzmarski et al., 2020). Indirect support for this possibility comes from our observation that microglia were initially activated in a time frame of days after of oligomeric Aβ(1–42) treatment, whereas we found evidence of astrocytic loss 6 months after treatment. Neuroinflammation may propagate or exacerbate AD (Heneka et al., 2015). Microglia engage in acute responses to pathogens and are directly activated by oligomeric forms of Aβ(1–42)(Paranjape et al., 2012). They bind to the peptide by means of cell surface receptors and dismantle it through endolysosomal and enzymatic pathways (Bradshaw et al., 2013; Griciuc et al., 2013; Wang et al., 2015), thereby abrogating the patholological effects of Aβ(1–42) by reducing its intracerebral titre (Jiang et al., 2014). This neuroprotective effect is short-lived however, and prolonged exposure to Aβ-(1–42) results in a substitution of anti-inflammatory M2 microglia to pro-inflammatory M1 microglia (Cherry et al., 2014; McGeer and McGeer, 2015). Thus, microglia may serve to exacerbate disease progression (Block et al., 2007; Dewapriya et al., 2013; Mosher and Wyss-Coray, 2014).

Deconstruction of astroglia complexity in dementia and AD has been reported in several studies (Rodríguez et al., 2009). The decreases in expression of GFAP that we detected 1 week after oligomeric Aβ(1–42) inoculation correspond to the acute and marked changes in morphology of astrocytes that occurred at this early time-point, from which brain tissue did not recover 6 months later. Treatment of Aβ(1–42) led to a reduction in number of main processes and arborisations of astrocytes. Studies in patients revealed that astrocytes of adult and healthy aged participants showed similar morphological complexity and that this differed from astrocytes of dementia patients (Senitz et al., 1995). Astrocytes secrete and re-uptake neurotransmitters (Mothet et al., 2005; Assefa et al., 2018), along with ions and neuromodulators (Verkhratsky and Nedergaard, 2014) and play an important role in neuroprotection (Arellano et al., 2016). They also internalize and degrade Aβ(1–42) (Wyss-Coray et al., 2003). The morphological changes in astroglia observed in our study may reflect an inability to provide support for synaptic functions that is reflected in the impoverishment of LTP that we observed. These findings also suggest that brain homeostasis is reaching an imbalance already at this relatively early stage of putative amyloidosis. Furthermore, the decrease in GFAP detected in our study may also reflect an acute loss of astrocytes. These changes can be expected to increase the vulnerability of the brain to Aβ(1–42) and other brain insults (Heneka et al., 2015), and may even propagate the disease (Birch, 2014; De Strooper and Karran, 2016). Our finding is also in line with reports of astroglial loss in other neurodegenerative disorders (Kovacs et al., 2017) and with reports of astroglial degeneration in transgenic models of AD (Olabarria et al., 2010; Yeh et al., 2011). That said, findings with regard to GFAP immunoreactivity in AD remain controversial (Calvo-Flores Guzmán et al., 2020).

An interesting link between the long-term loss of astrocytes and the progressive debilitation of LTP that we observed in the dentate gyrus, is the finding that astrocytes support adult neurogenesis in both rats (Sultan et al., 2015) and humans (Eriksson et al., 1998). The loss of GluN1 and thus NMDAR that we detected 6 months after treatment may in fact have been mediated by a reduction in newborn cells in the dentate gyrus (Sultan et al., 2015). Conversely, the loss of GluN1 may have contributed to a loss of newborn cells (Tashiro et al., 2006, 2007). Future studies could address this issue by analyzing the subgranular zone of the dentate gyrus using neurogenesis markers such as doublecortin or Ki-67 (von Bohlen und Halbach, 2011).

Taken together, these results show that a single perturbation of Aβ-homeostasis by means of oligomeric Aβ(1–42) elicits long-term and widespread effects on hippocampal function. Strikingly, this effect was observed in genetically non-manipulated healthy rats. A process is triggered that commences with a neuroinflammatory response in the initial days after the insult and is succeeded by changes in neuronal excitability and a loss of GluN1, the primary constituent of NMDAR (Stephenson, 2006), and morphological changes of astrocytes, that become evident 6 months later. Strikingly, the hippocampus does not recover from this perturbation: rather a progressive loss of hippocampal function occurs. We propose that a physiological insult that serves to temporarily overwhelm the effectivity of cellular systems to retain oligomeric Aβ-levels within their (non-pathological) physiological range, triggers pathophysiological processes that pave the way for the pathogenesis of amyloidosis. This putative vulnerability of the brain to transient elevations in oligomeric Aβ(1–42) may explain why so many disease and health states that impact on metabolism are proposed risk factors for AD (Mormino, 2014; Tarasoff-Conway et al., 2015). It could also explain why iatrogenic exposure to Aβ results in the development of AD in affected patients (Banerjee et al., 2024).

## Data availability statement

The raw data supporting the conclusions of this article will be made available by the authors, upon reasonable request.

## Ethics statement

The animal study was approved by the Landesamt fur Arbeitsschutz, Naturschutz, Umweltschutz und Verbraucherschutz (LANUV), North-Rhine Westphalia, Germany. The study was conducted in accordance with the local legislation and institutional requirements.

## Author contributions

MK: Investigation, Formal analysis, Writing – review & editing. T-HH: Visualization, Methodology, Writing – review & editing, Investigation, Formal analysis. HY: Writing – review & editing, Investigation, Formal analysis. OS: Visualization, Methodology, Writing – review & editing, Investigation, Formal analysis. JB: Writing – review & editing, Methodology, Investigation. UN: Formal analysis, Writing – review & editing, Methodology, Investigation. JC-K: Visualization, Writing – review & editing, Methodology, Investigation, Formal analysis. DM-V: Writing – original draft, Supervision, Resources, Funding acquisition, Conceptualization, Writing – review & editing, Methodology.
